# Drug-Induced Apnea in Children Admitted to Loghman Hakim Hospital, Tehran, Iran

**Published:** 2017

**Authors:** Narges GHOLAMI, Fathi ALWASABI, Fariba FARNAGHI

**Affiliations:** 1 Pediatric Department, Loghman Hakim Hospital, Shahid Beheshti University of Medical Sciences,Tehran, Iran; 2 Pediatric Medical Toxicology Department, Loghman Hakim Hospital, Shahid Beheshti University of Medical Sciences, Tehran, Iran

**Keywords:** Poisoning, Apnea, Pediatrics, Methadone

## Abstract

**Objective:**

Environmental hazards, including poisons, can cause irreparable effects and even fatal for children. Poisoning in children is common and serious, but often is preventable and treatable. This study aimed to evaluate the prevalence of drugs and chemical toxicity leading to apnea. In addition, we detected type of drug that induced apnea among children.

**Materials & Methods:**

In a retrospective cross-sectional study from Apr 2012 to Apr 2013, data of all hospitalized drug-induced Apnea in children were collected through hospital records.

**Results:**

The most common cause of drug toxicity was methadone opium, baclofen and heroin (74%,13%,5%,2%). The mortality rate was 3.1%; all of them due to methadone poisoning.

**Conclusion:**

There was a high prevalence of Methadone poisoning and apnea in children.

Methadone poisoning should be considered in apnea.

## Introduction

Children are vulnerable to numerous environmental hazards, including poisoning, which can have irreparable and even fatal effects on them. Although it is preventable and curable, but poisoning in children is one of the most common and hazardous causes of emergencies. Ingestion of a potentially toxic substance is a common reason for children and young people to attend the emergency ward or primary healthcare center.

Nowadays, the production of a variety of chemical, industrial and pharmaceutical synthetic materials has increased the probability of deliberate and accidental contaminations, it happens particularly among children. Poisoning is most common under 5 yr age group, also it is more frequent in boys (1).

The rate of child mortality caused by poisoning is variable. Drugs poisoning causes mortality and poisoning rates in undeveloped countries are four times more than other countries. The highest incidence of poisoning was reported in the lower middle socio-economic families (2).

A major cause of mortality or morbidity related to poisoning is hypoventilation and apnea that is a life-threatening and lethal condition.

The major challenge for us is to identify children at risk of severe symptoms and manage them appropriately.

Fortunately, mortality in a child from poisoning is not common and most substances are low toxicity.

However, there is some ingestion, which can result in serious problems and are potentially fatal in small doses in pediatrics (3).

Poisoning happens in 7% of all accidents in children under 5 yr, also it was led to death in over 5% of all pediatrics mortality in developing countries (4). 

Throughout poisoning should be regarded as one of the most important causes of child apnea in Iran due to its high prevalence.

The current study was performed in Loghman Hakim Hospital, Emergency Department of Pediatrics, Tehran, Iran, from Apr 2012 to Apr 2013. Our aim was to find out the prevalence of apnea in drug poisoning in children because of there are morbidity and mortality risks with some drugs can induce apnea in children and increased cases of poisoning among Iranian children. We investigated the type of drugs due to apnea in children as well.

## Materials & Methods

This study was an observational, retrospective, single center case series. We selected all children younger than 12 yr of age with apnea due to drugs and chemical poisoning who hospitalized during Apr 2012 to Apr 2013 in Loghman Hakim Hospital (a major center for poisoning treatment), Tehran, Iran. 

The Ethics Committee of the University approved the study. We excluded the patients with cardiorespiratory disease, a history of musculoskeletal diseases, probable aspiration of an external object, and history of trauma and drug allergy. Overall, 96 patients participated in this study. All of the patients had apnea caused by drug poisoning. All information was collected from the medical records written by hospital residents. We considered all date about kind and amount of drugs, time of poisoning, apnea and referring to hospital, age, sex and paraclinical findings.

**Data Analysis**

Data were analyzed using the SPSS version 20 statistical software (Chicago, IL, USA). P value <0.05 was considered significant.

## Results

Overall, 96 patients were enrolled of age range of 25 d to 12 yr old. There were 51 boys (53.1%) and 45 girls (46.9%). The most common cause of drug poisoning that led to apnea included methadone (74%) (Table 1). 

**Table 1:** The most common causes of drug poisoning that leads to apnea

Drug	Frequency	Percent
Methadone	**71**	**74.0**
**Opium**	13	13.5
Baclofen	**5**	**5.2**
Heroin	**2**	**2.1**
**Organophosphate toxin**	1	1.0
Unknown	**1**	**1.0**
Diphenoxylate	**1**	**1.0**
Scorpion stings	**1**	**1.0**
Tramadol	**1**	**1.0**
Total	**96**	**100.0**

The most common age of drug poisoning was from one to two years 22.9% followed by the age range from four to six years (14.6%). The most common drug poisoning leading to apnea was 53.1% among boys and 46.9% among girls. Opium was the most common cause of poisoning in infants younger than one month. However, in children between one month and six months of age, opium was the most common cause of poisoning (50%), followed by methadone (33.3%) and then baclofen (16.7%). In children between six months and one-yearold methadone was the most common cause of poisoning (53.8%), followed by opium (30.8%), and then tramadol and heroin (7.7%).

The most common method of drug poisoning was accidental (99%), just in one case, it was intentional. 

It occurred accidentally by parents (59.4%) and by the patient himself (40.6%). The interval between drug poisoning and apnea was 2.8 h. The minimum and maximum intervals were half an hour and 38 hours. 

Totally, 75 cases (78.1%) had apnea only once, 15 cases (15.6%) had two apnea episodes, and six cases (6.3%) had three apnea episodes. The maximum frequency of the occurrence of apnea was three times. 

Average of hospitalization in drug poisoning was about three days. Seizure due to drug poisoning leading to apnea happened in 18.8% of them. Cardiopulmonary resuscitation (CPR) was performed on 11.5% of the patients. 31.3% of the patients developed leukocytosis due to drug poisoning. Finally, 96.9% of patients had full recovery and three cases died (3.1%).

Overall, the most common cause of drug poisoning leading to apnea was methadone.

## Discussion

The rate of drug overdose deaths increased significantly for both sexes in the entire world, also there is poisoning in pediatrics but poisoning is one of the most common important critical conditions in children (5). Drug poisoning caused apnea is very important. Early diagnosis and immediate treatment are serious problems in poisoning led to apnea. Children are more vulnerable to various environmental risks, including poisoning, which can have irreparable and even fatal effects for them. Child poisoning is one of the most common and dangerous, although preventable, causes of emergency, and is considered as a common reason for referring to healthcare centers and being hospitalized. Numerous sources have discussed a wide range of poisoning induced deaths.

Although accidental poisoning happens at any age, it is much more common in children. It happens more in around the age of 2 yr, also boys are at more risk than girls are. Whereas drugs have not been stored in their suitable place or different container, 80%–85% of poisoning occurs at home (3, 6).

Methadone was the most important drug toxicity in childhood and apnea happened in 53.4% of cases ineffective breathing except apnea occurred in 62.1% and cyanosis 43.1% (4). Over half of apnea cases are caused by sedative and hypnotic drugs, including phenothiazine, barbiturates, and benzodiazepines, general anesthetics, painkillers, narcotic opioids, and drugs that affect on neuromuscular, autonomic ganglia, and cardiovascular functions(7).

Overall, 11150 children with drug poisoning hospitalized in Loghman Hakim Hospital, 37 cases (0.33%) died, 12 of whom (32.4%) had died before reaching the hospital. The ratio of boys to girls was 2:1, and the mean age of the patients was 5.3 yr old. All the poisonings were accidental and 37.8% of them occurred in the summer. Oral poisoning was the most common form (81%). In terms of the location, 70.2% of the patients came from Tehran suburbs or other towns, of whom 35% had arrived at the healthcare center more than six hours after the onset of poisoning. Poisonings that led to death included hydrocarbons 27%, CO poisoning, poisonous mushrooms and opium compounds that each accounting for 10.8%, organophosphate pesticides 8% and aluminum phosphide poisoning 8%, mercury vapor 5.4% and drugs 5.13% (8).

From Oct 2009 to Mar 2010 in this hospital, 16 boys and 15 girls were poisoned with methadone. The mean age of the patients was 55 months old (minimum four months and maximum 12 months old). Their parents poisoned all the patients either accidentally or mistakenly. The mean interval between the time of consumption and the onset of the symptoms was 1.53 h, which is a long time from the time of consumption to the onset of symptoms. 

Clinical symptoms included drowsiness 75%, miosis 68%, vomiting 61%, shallow and slow breathing 57%, and apnea 40% (9).

Children under three years old were more risky, most common substance was organophosphorus compounds (11.5%). Difficulty in breathing was present in 57.29% cases (10).

In one study, the frequency of clinical symptoms in opioid poisoning in 90 cases of children showed that apnea was 21.1% and cyanosis was 26.7%. Opioid poisoning and apnea were very important among Iranian children (11). 

According to the findings of this investigation, we should consider poisoning in children is serious. Families should know about drug poisonings lead to apnea. However, although it is preventable and curable it can be hazardous and lethal; therefore, parents must take their child to the nearest healthcare center when their children use toxic substance. Any child who refers to hospital with symptoms of apnea and cyanosis, the physicians must suspect drug poisoning, especially with methadone and opium; however, the physicians make early diagnosis and treat the patients on time. Firstly, in patients with severe respiratory depression or apnea, resuscitation by ventilation and oxygenation is necessary. According to this study methadone toxicity is common between Iranian children also it can lead to apnea. Doctors should try naloxone (antidote or substance that can counteract of opioid poisoning). We should know about the prevalence of methadone poisoning and apnea, and we seriously recommend that the use of this substance among families should be decreased. The toxic substance should not be easily accessible to children and families must keep them out of their reach. Addiction treatment centers should keep methadone in bottles with child-resistant cap and warning label and avoid putting methadone in drug glass or mineral water bottles. Factories should use warning design label on drugs and dangerous substances, which should be on national sign and parents and children should be warned and educated not to touch. Storing and keeping drugs in the house should be safe.

**In conclusion, **governments establish more poisoning centers for these patients. Appropriate prevention methods should be provided and people should be informed about drug poisoning better than before. 

## Conflict of interest

The authors declare that there is no conflict of interest.
